# Randomized phase II selection trial of FLASH and conventional radiotherapy for patients with localized cutaneous squamous cell carcinoma or basal cell carcinoma: A study protocol

**DOI:** 10.1016/j.ctro.2024.100743

**Published:** 2024-02-08

**Authors:** Rémy Kinj, Olivier Gaide, Wendy Jeanneret-Sozzi, Urania Dafni, Stéphanie Viguet-Carrin, Enea Sagittario, Magdalini Kypriotou, Julie Chenal, Frederic Duclos, Marine Hebeisen, Teresa Falco, Reiner Geyer, Patrik Gonçalves Jorge, Raphaël Moeckli, Jean Bourhis

**Affiliations:** a Department of Radiation Oncology, Lausanne University Hospital and University of Lausanne, CHUV, Rue du Bugnon 46, CH-1011 Lausanne, Switzerland; b Service of Dermatology, CHUV, Lausanne University Hospital, Lausanne, Switzerland; c Laboratory of Biostatistics School of Health Sciences University of Athens, Greece; d Center of Experimental Therapeutics (CTE), Lausanne University Hospital and University of Lausanne, Lausanne, Switzerland; e Institute of Radiation Physics, Lausanne University Hospital and University of Lausanne, Lausanne, Switzerland

**Keywords:** FLASH radiotherapy, Ultra high dose rate, Skin cancer, Squamous cell carcinoma, Basal cell carcinoma

## Abstract

**Background:**

Cutaneous basal cell carcinoma (BCC) and squamous cell carcinoma (SCC) are the most prevalent skin cancers in western countries. Surgery is the standard of care for these cancers and conventional external radiotherapy (CONV-RT) with conventional dose rate (0.03–0.06 Gy/sec) represents a good alternative when the patients or tumors are not amenable to surgery but routinely generates skin side effects. Low energy electron FLASH radiotherapy (FLASH-RT) is a new form of radiotherapy exploiting the biological advantage of the FLASH effect, which consists in delivering radiation dose in milliseconds instead of minutes in CONV-RT. In pre-clinical studies, when compared to CONV-RT, FLASH-RT induced a robust, reproducible and remarkable sparing of the normal healthy tissues, while the efficacy on tumors was preserved. In this context, we aim to prospectively evaluate FLASH-RT versus CONV-RT with regards to toxicity and oncological outcome in localized cutaneous BCC and SCC.

**Methods:**

This is a randomized selection, non-comparative, phase II study of curative FLASH-RT versus CONV-RT in patients with T1-T2 N0 M0 cutaneous BCC and SCC. Patients will be randomly allocated to low energy electron FLASH-RT (dose rate: 220–270 Gy/s) or to CONV-RT arm. Small lesions (T1) will receive a single dose of 22 Gy and large lesions (T2) will receive 30 Gy in 5 fractions of 6 Gy over two weeks.

The primary endpoint evaluates safety at 6 weeks after RT through grade ≥ 3 toxicity and efficacy through local control rate at 12 months. Approximately 60 patients in total will be randomized, considering on average 1–2 lesions and a maximum of 3 lesions per patients corresponding to the total of 96 lesions required. FLASH-RT will be performed using the Mobetron® (IntraOp, USA) with high dose rate functionality.

LANCE (NCT05724875) is the first randomized trial evaluating FLASH-RT and CONV-RT in a curative setting.

## Highlights

• Basal cell carcinoma (BCC) and squamous cell carcinoma (SCC) are the most prevalent skin cancers in western countries.
• In pre-clinical studies FLASH-RT induces a remarkable sparing of normal tissues, while preserving intact anti-tumor efficacy.
• LANCE is a randomized selection phase II trial of FLASH-RT versus CONV-RT for patients with T1-T2 N0 BCC or SCC of the skin.
• This is the first randomized trial evaluating FLASH-RT and CONV-RT in a curative setting.

## Introduction/rationale

Basal cell carcinoma (BCC) and squamous cell carcinoma (SCC) are the most prevalent skin cancer types in western countries, and represent more than 5 million cases annually in the United States with an increasing prevalence in Europe over the last decades [1]. Several therapeutic options are available such as surgical excision, conventional external radiation therapy (CONV-RT), cryotherapy, brachytherapy and topical treatments. While surgical excision is considered to be the primary curative treatment approach of cutaneous SCC and BCC, CONV-RT with conventional dose rate (0.03–0.06 Gy/sec) is recognized as an alternative treatment for well selected patients [2], [3], [4], [5]. In this frame, radiotherapy is generally delivered over a few weeks (2 to 8) which is not convenient for elderly patients who frequently present co-morbid conditions and have transfer issues. Moreover, CONV-RT routinely generates skin side effects for patients. According to CTCAE classification, a grade ≥ 2 radiation-induced acute toxicity such as dermatitis, mucositis and pain can be found in up to 20–70 % of patients after CONV-RT treatment and a late skin radio-necrosis in up to 6 % of patients after a single fraction treatment [6], [7], [8]. Other esthetical complications such as dyspigmentation and telangiectasia are also observed in 65–70 % of patients^9^.

The FLASH phenomenon, consisting in a remarkable sparing of healthy tissues with less side effects as compared to CONV-RT, was consistently described in preclinical setting [10]. For instance, one of the most striking result comes from the dose escalation experiment comparisons between CONV-RT and FLASH-RT on the skin of a mini-pig [11]. Single irradiation doses escalating from 22 Gy to 34 Gy were delivered to the same animal and at the same time. In comparison to 25 Gy delivered with low energy electron CONV-RT (dose rate ≈ 0.08 Gy/s), a dose of 34 Gy delivered with low energy electron FLASH-RT (dose rate ≈ 300 Gy/s) produced comparable results. Interestingly, severe late skin fibronecrosis was observed only with CONV-RT, assessing the sparing effect of FLASH-RT on the skin of the pig, especially from severe late effects. Comparable observations were collected across animal species (i.e., zebrafish, mice, mini-pig and cat), while providing an efficient anti-tumor effect [10]. The rational for clinical translation of FLASH-RT is based on the robustness and reproducibility of these results.

FLASH-RT has also been used successfully in a first patient at CHUV, as compassionate use, showing feasibility and safety of a 15 Gy single dose which was able to control a refractory skin lymphoma without generating significant side effects [12]. The patient was subsequently treated for two additional tumors at the same dose of 15 Gy, one with FLASH-RT and one with CONV-RT [13]. Late skin effects were evaluated at 24 months of follow up through clinical evaluation, photographs and biopsies, and there was no difference based on these criteria. This observation was compatible with the results of a comparison of FLASH-RT versus CONV-RT in mice skin. Soto *et al*, demonstrated a marked reduction of severe acute skin toxicity with FLASH –RT, which started to be detectable at 30 Gy single dose, whereas at lower dose levels (16 Gy, for example), the difference between FLASH and CONV were relatively minimal [14]. Consistently, a quantitative analysis gathered available in vivo data of normal tissue sparing of CONV-RT versus FLASH-RT single-fraction doses and converted these to a common scale using isoeffect dose ratios, referred to as FLASH-modifying factors (FMF). The results of the analysis revealed that the FLASH sparing effect markedly increased with dose, ranging from about 5 % (when dose < 10 Gy) to about 30 % (when dose > 25 Gy) [15]. The magnitude of the normal tissue sparing effect allowed by FLASH-RT represents another argument that justifies its clinical translation.

In this clinical trial, patients with localized T1-T2 N0 M0 cutaneous SCC and BCC who cannot undergo or decline surgical resection will be treated by a definitive curative radiotherapy and randomized between FLASH-RT or CONV-RT. Lesions will be divided into so called “small” and “large” volume groups. Small lesions are defined as T1 lesions (≤20 mm) whereas large lesions are defined as T2 lesions (>20 - ≤ 40 mm), according to the TNM UICC, 8th Edition. A single dose of 22 Gy will be delivered for lesions in “small volume” arm. A 22 Gy single dose FLASH therapy has already been tested in the ongoing CHUV-DO-0023-IMPulse-2020 dose escalation clinical trial (NCT04986696) and was shown to be safe when applied on skin metastases of melanoma of an overall volume ≤ 30 cc (data not yet published). The data from the ongoing IMPulse trial (NCT04986696) are re-assuring regarding the expected tolerance of the 22 Gy dose level in FLASH, and both SCC and BCC are less radioresistant than melanoma. For lesions in “large volume” arm, a dose of 30 Gy in 5 fractions (over 2 weeks) will be administered. It represents the minimal hypofractionated dose admitted for the treatment of skin lesions according to NCCN guidelines2, [4], [5], [16]. A grade ≥ 2 skin toxicity can be found in 20–70 % of patients (mainly radiation dermatitis, mucositis or pain) after CONV-RT in this range of dose [7], [17], [18].

In this context, our main hypothesis is that FLASH-RT will be well tolerated (primary endpoint) and efficient (hierarchically tested primary endpoint) in a population of patients with localized cutaneous SCC and BCC. LANCE trial represents the first curative intent FLASH trial.

## Design

This is an open-label single center randomized selection, non-comparative phase II study of FLASH-RT versus CONV- RT in patients with localized cutaneous SCC or BCC non-amenable to surgery, as per tumor board assessment.

**Fig. 1:**
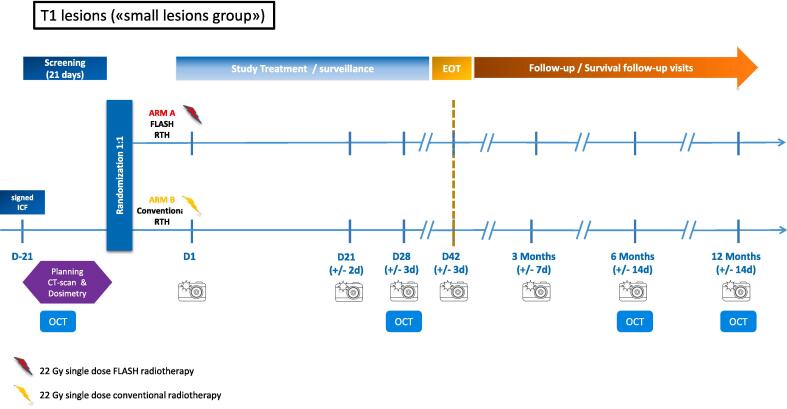
Overview of the study design for small volume lesions.

**Fig. 2:**
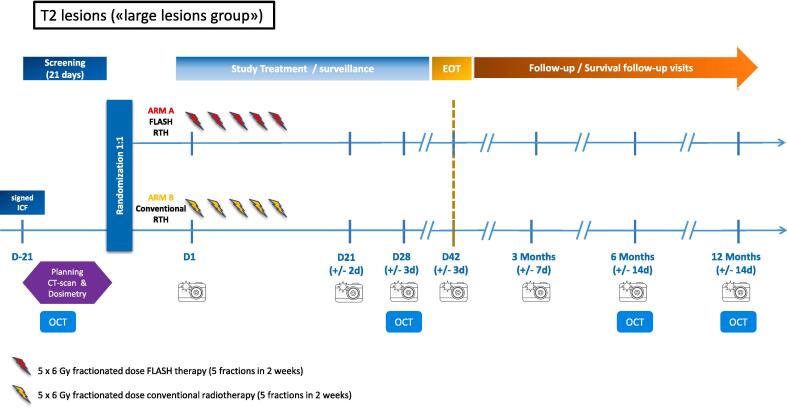
Overview of the study design for large volume lesions.

Patients will be randomly allocated to the FLASH-RT arm or to CONV-RT (according to standard guidelines) [4], [6], [16] treatment arm, as described below **(**Fig. 1**,** Fig. 2**)**:

*For T1 (small, ≤ 20 mm) lesions* ***:***

­ Arm A: 22 Gy single dose FLASH-RT
­ Arm B: 22 Gy single dose CONV-RT

For FLASH-RT arm, a 9 MeV electron FLASH-RT will be delivered in 10 pulses of 2.2 Gy each, delivered at a pulse repetition frequency of 90 Hz for a total delivery time of 100 ms, corresponding to a mean dose rate of 220 Gy/s.

For CONV-RT arm, a 10 MeV electron CONV-RT will be delivered at a conventional dose rate (approximately 2–8 Gy/min) with a clinical linac. A bolus of 5–10 mm will be used to compensate for the difference in beam penetration between the FLASH-RT and CONV-RT arms.

*For T2 (large, >20 – ≤ 40 mm) lesions* ***:***

­ Arm A: 5 x 6 Gy fractionated dose FLASH-RT (5 fractions in less than 2 weeks)
­ Arm B: 5 x 6 Gy fractionated dose CONV-RT (5 fractions in less than 2 weeks)

For FLASH-RT arm, a 9 MeV electron FLASH-RT will be delivered in 3 pulses of 2 Gy each, delivered at a pulse repetition frequency of 90 Hz for a total delivery time of 22 ms, corresponding to a mean dose rate of 270 Gy/s.

For CONV-RT arm, the same beam parameters as in the small CONV-RT arm will be used with a dose adaptation.

Inclusion and exclusion criteria are shown in Table 1.

**Table 1:** Inclusion and exclusion criteria.

Inclusion criteria	Exclusion criteria
Signed study Informed Consent Form Karnofsky Performance Status (KPS) ≥ 60 % Age ≥ 60 years Patients with histologically proven cSCC or BCC Patients requiring radiotherapy treatment according to dermato-oncology tumor board: patients who cannot undergo surgical procedure or patients who decline surgical resection and/or anatomical locations where surgery can compromise function or cosmesis.T1-T2 N0 M0 lesions with a small (T1; lesion ≤ 2 cm in diameter) or large (T2; 2 cm < lesion ≤ 4 cm) volume (TNM UICC, 8th Edition) Lesions should be at least 4 cm apart if treated with 2 different modalities (including surgical treatment of lesions). Lesions should not be located on the face, except on the forehead, above a line situated 1 cm above the eyebrows. Lesions located on the scalp can be treated	Previous radiotherapy in the treated area Concomitant auto-immune disease with skin lesions Concomitant use of radio-sensitizer drug Cognitive disorders, not compatible with the signature of informed consentCurrent, recent (within 10 days prior start of study treatment), or planned participation in an experimental drug study (before EOT visit)Concomitant use of systemic oncological treatment for a cancer other than the skin cancer (s)

## Objectives and endpoints

### Objective

The aims of the study are to describe and compare the toxicity and efficacy of FLASH-RT to CONV-RT (according to the dose per lesion size) through a randomized phase II selection study in patients presenting localized cutaneous SCC or BCC requiring a radiotherapy treatment.

### Endpoints

Primary endpoint

• Safety measured by ≥ grade 3 skin toxicity up to 6 weeks after radiotherapy

Hierarchically tested primary endpoint.

• Hierarchically tested efficacy measured by local control rate

Safety will be measured by ≥ grade 3 skin toxicity as defined by Common Terminology Criteria for Adverse Events (CTCAE v5.0) [8]. Local control rate is censured by any histologically proven local relapse into the initial treatment field up to one year post randomization.

Secondary endpoints

• Acute side effects in the treatment field
• Late side effects in the treatment field (≥3 months)
• Blinded Imaging Central Review (BICR) of photographs evaluating both tumor response and “in radiation field” normal tissues reaction around the treated tumors
• Optical coherence tomography (OCT) examination of the irradiated skin will be compared to the normal non-irradiated skin at baseline, 4 weeks, 6 months, and 12 months post-treatment

## Statistical design

This is a randomized phase II selection design with two treatment arms, where each individual arm is structured as an independent single-arm phase II study [19]. In this setting, randomization is primarily for the purpose of reducing various types of bias, such as patient selection bias and controlling for known or unknown baseline imbalances between arms.

Each arm will be evaluated separately, with hierarchical testing of 1. Safety and 2. Efficacy as measured by local tumor control rate.

### Safety evaluation (for each study arm separately)

The safety will be evaluated based on a Simon’s two-stage design with targeted rate of skin toxicity of grade ≥ 3 (π^s^; number of lesions with at least one skin toxicity of grade 3 or higher, up to 6 weeks after radiotherapy, among all treated lesions) less than 10 %, and considering as unacceptable, a rate of 25 %. That is, we test the null hypothesis H_0_: π^s^_0_ ≥ 25 % versus the alternative H_1_: π^s^_1_ < 25 %, evaluated at π^s^_1_ = 10 %.

A sample size of 48 lesions (of small or large size) per study is needed to test the above hypothesis, at a one-sided 5 % type I error and power of 80 %.

If at the interim evaluation (first stage of Simon’s two-stage design), more than 10 lesions in the first 13 evaluated lesions do not present with skin toxicity of grade ≥ 3, the study arm will continue to accrual completion. If more than 40 out of the 48 lesions, do not present with skin toxicity of grade ≥ 3, then the corresponding RT treatment will be considered safe and the evaluation of efficacy for this study arm will proceed.

### Hierarchically tested: Efficacy evaluation (for each study arm separately)

Regarding efficacy in each of the two arms, if the safety question is satisfied then the hierarchically tested primary endpoint local tumor control rate (π^e^; number of lesions without relapse over the total number of randomized lesions) will be evaluated in the 48 lesions in the specific arm, assuming that the targeted local tumor control rate is ≥ 90 % [9].

An exact Binomial design for single proportion will be used, at a one-sided 5 % type I error, and a power of 80 %, for testing a local control rate ≤ 75 % versus a local control rate of at least 90 %, on the 48 lesions in the corresponding arm. That is, we test the null hypothesis H_0_: π^e^_0_ ≤ 75 % versus the alternative H_1_: π^e^_1_ > 75 %, evaluated at π^e^_1_ = 90 %.

### Number of participants with rationale

Approximately 60 patients in total will be randomized, considering on average 1–2 lesions and a maximum of 3 lesions per patients (a mix of small and large lesions can be treated on a same patient), corresponding to the total of 96 lesions required (48 per study arm) (Fig. 1**,** Fig. 2). Lesion sample size and power calculations have been performed in EAST v6.5, Cytel Inc, Massachusetts, USA.

### Investigational medical device

Clinical translation of FLASH therapy requires appropriate irradiation device able to deliver the dose of irradiation in milliseconds instead of minutes that are commonly used for CONV-RT. In this clinical trial, we will use a Mobetron® (IntraOp, USA) with high dose rate (HDR) functionality for superficial skin cancer treatments [20]. The device has been commissioned for 6 and 9 MeV HDR electron beams, only 9 MeV beam will be used in the trial [21]. The FLASH effect has been reproduced on that device with HDR functionality by two different centers [22]. Dose calculation will be performed based on percentage depth dose and dose profile.

The pulse structure that will be used for the study is composed of 3–10 pulses of 2–2.2 Gy each, delivered at a pulse repetition frequency of 90 Hz for a total delivery time of 22–100 ms, corresponding to a dose rate of 220–270 Gy/s depending on the delivered dose. The target volume corresponds to the visualized skin lesion with a 5 mm margin. For both arms, collimators with equal diameters (2, 3, 4 or 5 cm) will be chosen regarding the target volume and define the treatment field.

All operators of the Mobetron® with HDR functionality will be mandatory trained on the machine. Training will be supervised and validated by the Head of Radiotherapy Medical Physics group at CHUV.

### Planned timeline

The estimated duration of recruitment is 30 months with a completion date in the end of 2026, follow up is 12 months after last treated patient. A possible premature closure is possible in case of unforeseen toxicity at interim analysis, in case of ≥ 3 grade ≥ 3 in the first 13 evaluated lesions.

Ethical considerations

LANCE trial received ethical approval from Cantonal Ethics Committee (CER-VD), SwissEthics, Swissmedic and Federal Office of Public Health (OFSP). The Investigator is responsible for the conduct of the trial and will ensure that the trial is performed in accordance to the protocol and with principles enunciated in the current version of the Declaration of Helsinki [23], the guidelines of Good Clinical Practice (GCP) issued by ICH [24], the European Regulation on medical devices 2017/745 [25] and the ISO Norm 14,155 [26] and ISO 14971, the Swiss Law and Swiss regulatory authority’s requirement [27], [28], [29]. The competent ethics committee and Swissmedic will receive an annual safety report and be informed about study stop/end in agreement with local requirements. The trial is registered on Kofam.ch and Clinicaltrial.gov (NCT05724875).

## Funding

The study is granted by ISREC Foundation, Biltema Foundation and Fondation pour le soutien du développement et de la recherche en oncologie (FSRDO).

## CRediT authorship contribution statement

**Rémy Kinj:** Conceptualization, Writing – original draft, Writing – review & editing, Methodology, Investigation. **Olivier Gaide:** Conceptualization, Writing – review & editing, Methodology, Investigation. **Wendy Jeanneret-Sozzi:** Writing – review & editing, Investigation. **Urania Dafni:** Conceptualization, Data curation, Formal analysis, Methodology, Writing – review & editing. **Stéphanie Viguet Carrin:** Project administration, Software, Writing – review & editing. **Enea Sagittario:** Project administration, Software, Writing – review & editing. **Magdalini Kypriotou:** Project administration, Software, Writing – review & editing. **Julie Chenal:** Project administration, Software, Writing – review & editing. **Fredecric Duclos:** Investigation. **Marine Hebeisen:** Investigation. **Teresa Falco:** Writing – review & editing, Investigation. **Reiner Geyer:** Project administration, Software, Writing – review & editing, investigation. **Patrick Goncalves Jorge:** Project administration, Software, Writing – review & editing, investigation. **Raphael Moeckli:** Project administration, Software, Writing – review & editing, investigation. **Jean Bourhis:** Conceptualization, Methodology, Writing – original draft, Writing – review & editing, Investigation, Supervision, Funding acquisition.

## Declaration of competing interest

The authors declare the following financial interests/personal relationships which may be considered as potential competing interests: JB reports advisory role for Roche, BMS, MSD, Astra-Zeneca, Debiopharm, Nanobiotix, Merck and Mevion, Grant research with IntraOp, PMB and Theryq. RM receives a research grant from Accuray. All other authors have nothing to declare.
